# Readmissions and Death after ICU Discharge: Development and Validation of Two Predictive Models

**DOI:** 10.1371/journal.pone.0048758

**Published:** 2012-11-07

**Authors:** Omar Badawi, Michael J. Breslow

**Affiliations:** 1 Research & Product Innovation, Philips Healthcare, Baltimore, Maryland, United States; 2 Department of Pharmacy Practice and Science, University of Maryland School of Pharmacy, Baltimore, Maryland, United States; University of Sao Paulo Medical School, Brazil

## Abstract

**Introduction:**

Early discharge from the ICU is desirable because it shortens time in the ICU and reduces care costs, but can also increase the likelihood of ICU readmission and post-discharge unanticipated death if patients are discharged before they are stable. We postulated that, using *e*ICU® Research Institute (*e*RI) data from >400 ICUs, we could develop robust models predictive of post-discharge death and readmission that may be incorporated into future clinical information systems (CIS) to assist ICU discharge planning.

**Methods:**

Retrospective, multi-center, exploratory cohort study of ICU survivors within the *e*RI database between 1/1/2007 and 3/31/2011. Exclusion criteria: DNR or care limitations at ICU discharge and discharge to location external to hospital. Patients were randomized (2∶1) to development and validation cohorts. Multivariable logistic regression was performed on a broad range of variables including: patient demographics, ICU admission diagnosis, admission severity of illness, laboratory values and physiologic variables present during the last 24 hours of the ICU stay. Multiple imputation was used to address missing data. The primary outcomes were the area under the receiver operator characteristic curves (auROC) in the validation cohorts for the models predicting readmission and death within 48 hours of ICU discharge.

**Results:**

469,976 and 234,987 patients representing 219 hospitals were in the development and validation cohorts. Early ICU readmission and death was experienced by 2.54% and 0.92% of all patients, respectively. The relationship between predictors and outcomes (death vs readmission) differed, justifying the need for separate models. The models for early readmission and death produced auROCs of 0.71 and 0.92, respectively. Both models calibrated well across risk groups.

**Conclusions:**

Our models for death and readmission after ICU discharge showed good to excellent discrimination and good calibration. Although prospective validation is warranted, we speculate that these models may have value in assisting clinicians with ICU discharge planning.

## Introduction

Prolonged duration of stay in the intensive care unit (ICU) is costly, is stressful for patients and families, reduces the number of beds available for other patients, and can increase risk for iatrogenic and nosocomial complications. [1] ICU daily care costs are 2–3 fold higher than costs on general medical – surgical wards, reflecting both higher staffing ratios and greater resource consumption. [2] Strategies to decrease ICU length of stay (LOS) can improve patient throughput and increase the number of patients that can be cared for in capacity-constrained ICUs. Patients also benefit from shorter exposure to the disruptive ICU environment, and may have less sleep disruption caused by intensive monitoring and frequent audible alarms. [3], [4].

Early discharge from the ICU is not without risk. If patients requiring high intensity care are discharged before they can be safely cared for in a lower acuity care environment, they are at risk for both complications and delayed recognition of clinical deterioration. The former can result in the need for unplanned ICU readmission; the latter can result in patient death. Patients readmitted to ICUs have higher risk-adjusted mortality and lengths of stay. [5]–[7] The actual increases in mortality and LOS may be modified by contextual factors such as bed occupancy rates and patient inflow volumes. [8], [9] In addition, ICU readmission also places stress on patients and families.

Determining who is ready for ICU discharge is a daily challenge for ICU leaders, especially in units with high occupancy rates. Traditionally these decisions are made by attending physicians, in collaboration with other members of the ICU care team. [10] Due to the highly subjective nature of these decisions, there is considerable variability in determining discharge readiness. [11] There are few data on why patients deteriorate after ICU discharge, and differentiating problems present at the time of discharge from those that originate after discharge oftentimes is not possible. In the absence of this information it is generally assumed that the shorter the time between discharge and readmission or death, the more likely the patient was not ‘ready’ to be discharged from the ICU. As a result, 48 hours has historically been considered the primary timeframe for evaluating the quality of ICU discharges. [12].

Several studies have evaluated post-discharge patients and identified variables that predict these complications. [6]–[7], [13]–[22] Previously identified predictors of death or readmission include duration of ICU LOS, Glasgow Coma Scale (GCS) score at the time of ICU discharge, mean arterial blood pressure and ICU admission source. [15] Some investigators have attempted to create decision support tools to assist in discharge readiness assessment. Zimmerman and co-workers utilized the probability of next day risk for life support as a proxy means for determining discharge readiness. [21] They reported that the greatest risk factors were the current day’s therapy and the Acute Physiology and Chronic Health Evaluation III (APACHE®) score (Cerner Corp, Kansas City, MO). The SWIFT score, which focused on patients with readmission or death within 1 week of ICU discharge, demonstrated moderate discrimination, although significantly higher than the day of discharge APACHE III score. [13] The present study leverages a large and rich database of over 1,500,000 critically ill patients cared for in several hundred United States ICUs. [23], [24] The objective of this study was to attempt to develop robust predictive models, when embedded in electronic clinical information systems (as the ICU Discharge Readiness Score), might have value as a decision support tool to assist ICU leaders in making discharge decisions. We hypothesized that the very large sample size and the inclusion of several hundred different ICUs would provide sufficient power and heterogeneity to enable the creation of generalizable predictive algorithms.

## Methods

This was a retrospective, multi-center, exploratory cohort study utilizing ICU patients in the *e*ICU® Research Institute (*e*RI) database with a complete hospitalization between January 1, 2007 and March 31, 2011. Detailed descriptions of the *e*RI database are provided elsewhere. [23], [24] Although specific data on hospital demographics were not included in the analytic dataset, the *e*RI database represents geographically dispersed hospitals, with approximately 50% being teaching hospitals, 34% with over 500 beds and 12% with less than 100 beds. [11], [13] This study was exempt from IRB oversight as there were no patient interventions due to the retrospective design and the security schema for the *e*RI database was analyzed and re-identification risk was certified (45 Code of Federal Regulations 164.514(b)(1) and 45 C.F.R. 164.514(b)(1)(i); HIPAA Certification #80503C) as meeting safe harbor standards by Privacert, Inc. (Pittsburgh, PA). All patients discharged from participating ICUs were included in the analysis unless any of the exclusion criteria were met. Patients were randomized in a 2∶1 fashion to development and validation cohorts. Patients with the following conditions were excluded from the analysis: ICU LOS of less than 4 hours; age <16 years; expired in the ICU; discharge location of transfer to another ICU or locations external to the hospital; and the presence of a “do not resuscitate” (DNR) or “comfort measures only” (CMO) order at ICU discharge. Due to the retrospective design, all discharges from the ICU were made at the discretion of the attending physician. The primary study objective was to develop two predictive models; one predicting death and the other predicting readmission within 48 hours of ICU discharge.

Differences in baseline characteristics between the development and validation cohorts were assessed with Pearson Chi-square for categorical data, student t-test for normally distributed continuous variables and Wilcoxon-rank-sum for non-normally distributed continuous variables. Using the development cohort, associations between predictor variables and the primary outcomes (death or readmission) were evaluated using multivariable logistic regression. Continuous variables were assessed for non-linear relationships with the primary outcome using locally weighted scatterplot smoothing (LOWESS). Non-linear relationships were handled via introduction of spline terms (knots) or categorizing continuous variables. Spline terms were introduced to create intervals of existing linear relationships which changed slopes at knots designated by visual inspection of the locally weighted scatterplot smoothing.

59 different variables were evaluated for inclusion in the predictive models for post-discharge death and readmission within 48 hours of ICU discharge based upon clinician assessment of possible relevance. Variables included: patient demographics, ICU admission diagnosis, admission severity of illness determined by the APACHE score, intensive care interventions, complications occurring during the ICU stay, and laboratory and physiologic variables from the last 24 hours of the ICU stay. A complete list of variables included is described in Table S1. In order to reduce the number of diagnoses used in the model the 407 unique APACHE admission diagnoses were consolidated into 26 diagnosis groups (Table S2). Diagnoses were first ranked by prevalence and then grouped according to pathophysiology, with all rare diagnoses unrelated to newly created diagnosis groups categorized together as “Other”. The number of patients in the development cohort with original data available is presented for each predictor. To reduce the potential for introducing bias due to missing data patterns, multiple imputation was used for all predictors included in the final model unless specified in Table S1. [25]–[27] Multivariable regression was used to create five imputations using chained equations (ICE) via the “mi impute chained” command in Stata 12 (StataCorp. 2011. Stata Statistical Software: Release 12. College Station, TX: StataCorp LP).

A combination of methods was used to identify the initial set of possible predictors of death or readmission within 48 hours of ICU discharge. These included prior literature, clinical knowledge and forward and backward step-wise multivariable logistic regression. [5], [13], [18], [22] Variables were included in the step-wise regressions if the difference in log likelihood between the null versus extended models produced a p-value <0.05 using the log-likelihood ratio test for readmission and a p-value <0.01 for death. A more conservative threshold was used for the risk of death model due to the greater number of variables significantly associated with the outcome. All variables identified by these means were included in the initial development models. These were reduced to more parsimonious models by examining the difference in area under the receiver operating characteristic curve (auROC) between the null and extended models. As the ultimate goal is to develop predictive models that can be embedded in electronic clinical decision support tools, inclusion in the final models was based on balancing model performance against availability of data in the clinical information system. Therefore, some variables which did not tangibly improve model performance were excluded even if a significant association with the endpoint existed. As opposed to epidemiology studies seeking to quantify the relationship between specific variables and outcomes, the focus of predictive modeling research is on model accuracy. Therefore, collinearity between variables was allowed (e.g. use of both average heart rate and highest heart rate) because it improved performance of the predictive models. However, allowing multiple related variables makes it difficult to clinically interpret the adjusted odds ratios. The unadjusted odds ratio (OR) is presented for each of the covariates included in the final models.

The primary analytic measure used to assess model discrimination was the auROC for the development and validation cohorts. The Hosmer-Lemeshow goodness-of-fit test along with visual inspection of calibration curves were used to assess calibration across deciles of risk. The median and range for the discrimination and calibration across the five imputed datasets was reported. Performance of the models also were assessed in different ICU patient types (e.g., medical, surgical) and across hospital size and teaching status by comparing actual to predicted event rates within groups. A secondary validation step was performed to simulate expected real-time performance as a clinical decision support tool in the ICU. Fitted values (predicted probabilities) were determined using patients’ clinical data at 24 hour intervals prior to ICU discharge. The median and interquartile range (IQR) of the fitted values were calculated for up to 4 days prior to ICU discharge for 3 patient groups: a) patients discharged without an event of interest within 48 hours; b) patients discharged with an event within the subsequent 48 hours; and c) patients who did not survive the ICU stay. A linear regression (with a robust variance estimator clustered by patient) of the fitted values across the last 4 ICU days was calculated for each of these groups to describe trends in risk over this time period. All statistics were performed using Stata 12 (StataCorp. 2011. Stata Statistical Software: Release 12. College Station, TX: StataCorp LP).

## Results

**Figure 1:**
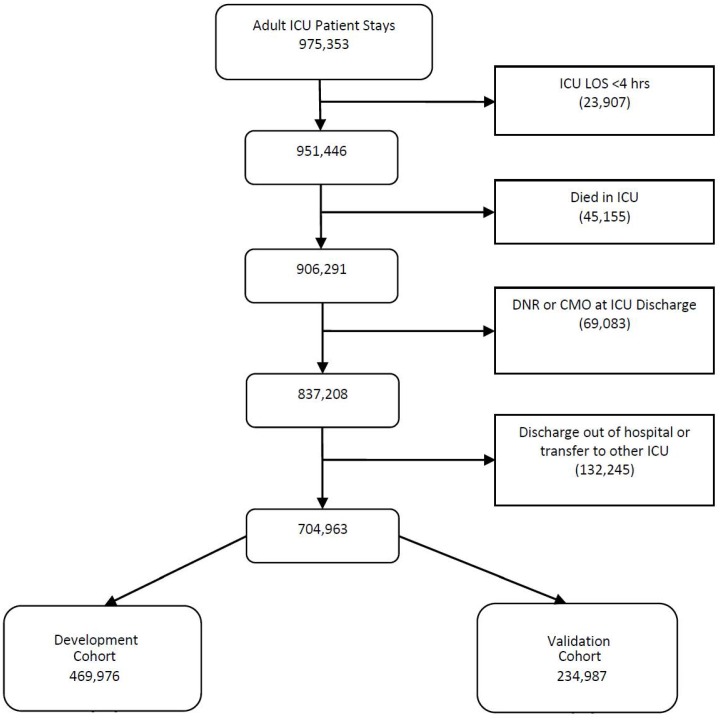
Cohort flow diagram. DNR  =  Do Not Resuscitate; CMO  =  comfort measures only; LOS  =  length of stay.

704,963 patients from 32 health systems were included in the final cohort, representing 219 hospitals and 402 ICUs from throughout the United States *e*RI database. 469,976 patients were randomized to the development set and 234,987 to the validation cohort. Figure 1 details the number of patients excluded from the cohort. Of the 704,963 patients in the cohort, 17,874 (2.54%) had a readmission and 6,492 (0.92%) died within 48 hours of discharge from the ICU. 499 (0.07%) patients experienced both a readmission and death within 48 hours of ICU discharge. The general characteristics of the development and validation cohorts are presented in Table 1. Although the median LOS appears low for this cohort of ICU survivors, the mean and standard deviation for the ICU and hospital LOS were 3.0 (±3.6) days and 4.3 (±5.0 days) respectively.

**Table 1:** Patient characteristics for patients included in models predicting death and readmission after ICU discharge.

	Development Cohort	Complete Data (N)	Validation Cohort	Complete Data (N)	P-Value
**Gender, % Male** a	54.1	469,976	53.9	234,987	0.56
**Race, % White** a	72.7	469,976	72.8	234,987	0.85
**Age, mean (SD)** b	62.1 (17.0)	469,976	62.1 (16.9)	234,987	0.49
**BMI, mean (SD)** b	29.0 (8.3)	424,316	29.0 (8.3)	212,085	0.86
**ICU Discharge Location, % Floor** a	83.3	469,976	83.4	234,987	0.26
**APACHE IV Score, median (IQR)** c	47(35–62)	342,168	47(35–62)	170,654	0.84
**Hospital Mortality, # (%)** a	14,472 (3.1)	469,976	7,275 (3.1)	234,987	0.72
**ICU LOS, median days (IQR)** c	1.88(1.04–3.35)	469,976	1.89(1.05–3.35)	234,987	0.08
**Hospital LOS, median days (IQR)** c	2.50(1.39–5.05)	469,976	2.51(1.39–5.05)	234,987	0.30
**Death within 48 hours, # (%)** a	4,389(0.93)	469,976	2,103(0.89)	234,987	0.11
**Readmission within 48 hours, # (%)** a	11,925(2.54)	469,976	5,949(2.53)	234,987	0.89
**ICU Type, # (%)** a		469,976		234,987	0.55
**Cardiac Medical**	91,364 (19.4)		45,934 (19.6)		
**Cardiovascular or Cardiothoracic Surgery**	51,367 (10.9)		25,566 (10.9)		
**Medical**	37,434 (8.0)		18,893 (8.0)		
**Mixed Medical-Surgical**	231,597 (49.3)		115,702 (49.2)		
**Neurological**	22,225 (4.7)		10,963 (4.7)		
**Surgical**	34,787 (7.4)		17,360 (7.0)		
**Trauma**	1,202 (0.3)		569 (0.2)		

a P-value calculated using Pearson Chi-square.

b P-value calculated using student t-test.

c P-value calculated using two-sample Wilcoxon rank-sum (Mann-Whitney) test.

APACHE  =  Acute Physiology and Chronic Health Evaluation IV Score; BMI  =  Body Mass Index; ICU LOS  =  ICU length of stay in days; IQR  =  Interquartile Range; SD  =  Standard Deviation.

Of the 59 variables initially analyzed in the development set, 26 and 23 were retained in the final models for death and readmission, respectively. Tables 2 and 3 show the unadjusted ORs of variables used in each model. Eight variables present on admission to the ICU were retained in at least one of the final models; admission diagnosis (including whether related to elective or emergent surgery), admission source, unit type, ICU visit number, age, and BMI. The remaining predictors came from data obtained during the last 24 hours of the ICU stay. Numerous continuous predictors had non-linear associations with the study outcomes, and were handled with spline terms or by categorizing in the final model. For example, for average heart rate over the last 24 hours, the odds of death decreased by 6% for each increase in beat per minute (bpm) up to 60 bpm, but increased by 5% for each bpm above 60. The relationship between the independent variables and the two separate outcomes were not necessarily consistent. In general, the relationships between independent variables and outcome were stronger for predicting death than readmission. In some cases there were clinically different relationships, as observed with average diastolic blood pressure over the prior 24 hours, where the odds of death increased by 8% for each mmHg over 100 mmHg while readmission risk was unchanged above 82 mmHg.

**Table 2:** Unadjusted odds ratios for variables retained in the final logistic regression model predicting death within 48 hours of ICU discharge in development cohort.

Variable	Unadjusted Odds Ratio	95% CI	Patients with Complete DataN = 469,976
**Admission Characteristics**			
** Age (per year)**	1.04	1.04–1.04	469,976 (100%)
** BMI (per kg/m^2^)** a			424,316 (90.3%)
** BMI <26**	0.92	0.90–0.93	
** BMI >26**	0.99	0.98–1.00	
**Operative Diagnosis (True)**	0.30	0.27–0.33	469,976 (100%)^b^
** Elective Surgery (True)**	0.25	0.22–0.28	469,976 (100%)^b^
**ICU Interventions**			
** ICU Length of Stay (per day)**			469,976 (100%)
** ICU LOS <1 day**	0.10	0.08–0.12	
** ICU LOS 1–2 days**	1.93	1.74–2.13	
** ICU LOS >2 days**	1.07	1.06–1.08	
** Ventilation Status**			469,399(99.9%)
** Spontaneous Breathing (reference)**	1.00	–	
** Non-Invasive Ventilation**	4.50	4.01–5.05	
** Spontaneous- Tenuous Ventilation**	5.36	4.91–5.85	
** Ventilation- Chronic Dependency**	4.70	3.07–7.18	
** Ventilation- Rapid Wean/Extubation**	5.99	3.95–9.09	
** Ventilation- Daily Extubation Trial**	33.13	30.46–36.02	
** Ventilation- No Daily Extubation Trial**	70.35	65.58–79.08	
**Last Day Labs**			
** Acid Base Status** c			469,976 (100%)
** Normal (reference)**	1.00	–	
** Acidosis**	6.13	5.68–6.62	
** Alkalosis**	2.72	2.47–2.99	
** Average Serum Lactate (per mMol/L)** ***^d^***			469,976 (100%)
**<0.5 mMol/L**	2.74	2.22–3.37	
**>0.5 mMol/L**	1.45	1.41–1.48	
** Maximum Serum Creatinine**			430,885 (91.7%)
**<1 mg/dL**	0.55	0.42–0.72	
**1–2 mg/dL**	6.30	5.70–6.96	
**>2 mg/dL**	0.91	0.89–0.93	
** Average White Blood Cell Count (per 10^3^/µL)**			418,283 (89.0%)
** <4×10^3^/µL**	0.46	0.43–0.50	
** 4–9×10^3^/µL**	1.11	1.07–1.14	
** >9×10^3^/µL**	1.08	1.08–1.08	
** Average Serum Glucose (per mg/dL)** ***^e^***			445,317 (94.8%)
**<100 mg/dL**	0.97	0.96–0.97	
**100–180 mg/dL**	1.01	1.01–1.01	
**>180 mg/dL**	1.00	1.00–1.00	
** Glucose Variability** ***^f^***	2.01	1.67–2.42	445,317 (94.8%)
**Last Day Physiology**			
** Average Diastolic Blood Pressure (per mmHg)**			456,617 (97.2%)
**<60 mmHg**	0.92	0.92–0.93	
**60–100 mmHg**	0.98	0.98–0.99	
**>100 mmHg**	1.08	1.04–1.12	
** Diastolic Blood Pressure Variability** ***^f^*** ** (per 100 units)**			455,808 (97.0%)
** <7.5**	0.95	0.82–1.10	
** 7.5–15**	1.06	1.03–1.08	
** >15**	1.06	1.06–1.06	
** Average Heart Rate (per bpm)**			464,494 (98.8%)
**<60 bpm**	0.94	0.91–0.96	
**>60 bpm**	1.05	1.05–1.05	
** Minimum Heart Rate (per bpm)**			464,494 (98.8%)
** <55**	0.90	0.89–0.90	
** 55–70**	1.02	1.01–1.03	
** >70**	1.05	1.04–1.05	
** Heart Rate Variability** ***^e^*** ** (per 100 units)**			463,780 (98.7%)
** <2.2**	0.98	0.83–1.16	
** 2.2–8**	0.87	0.85–0.89	
** >8**	1.10	1.09–1.10	
** Average Mean Arterial Pressure (per mmHg)**			456,193 (97.1%)
**<80 mmHg**	0.91	0.91–0.91	
**80–105 mmHg**	1.00	1.00–1.01	
**>105 mmHg**	1.01	1.00–1.03	
** Minimum Mean Arterial Pressure (per mmHg)**			456,193 (97.1%)
**<70 mmHg**	0.95	0.95–0.95	
**70–125 mmHg**	1.01	1.00–1.01	
**>125 mmHg**	1.03	0.90–1.17	
** Average Respiratory Rate (per bpm)**			452,882 (96.4%)
**<18 bpm**	0.93	0.91–0.95	
**>18 bpm**	1.17	1.16–1.18	
** Average SpO_2_ (per unit)**			458,080 (97.5%)
** <96%**	0.79	0.78–0.80	
** >96%**	1.14	1.10–1.17	
** Minimum SpO_2_ (per unit)**			458,080 (97.5%)
** <93%**	0.93	0.93–0.94	
** >93%**	1.04	1.01–1.07	
** SpO_2_** c **Variability (per 100 units)**			455,734 (97.0%)
** <1**	0.53	0.42–0.66	
** >1**	1.34	1.33–1.35	
** Average Systolic Blood Pressure (per mmHg)**			456,637 (97.2%)
**<115 mmHg**	0.92	0.92–0.92	
**115–145 mmHg**	1.00	1.00–1.00	
**>145 mmHg**	1.03	1.02–1.03	
** Systolic Blood Pressure Variability** ***^f^*** ** (per 100 units)**			455,832 (97.0%)
** <6**	0.75	0.66–0.85	
** >6**	1.14	1.14–1.15	
** Most Recent GCS Score (per unit)**	0.63	0.63–0.64	264,657 (56.3%)

a BMI considered missing if <10 or >125.

b 47,231 (10.0%) missing and considered to be “False”.

c Based on average pH value. pH missing in 358,425 (76.3% of patients).

Normal  =  pH between 7.34 and 7.44 or absence of data; Acidosis  =  pH lower than 7.34; and Alkalosis  =  pH greater than 7.44. *d.* 441,635 (94.0%) serum lactate values missing. Absence of a serum lactate value was treated as 0. *e*. Glucose considered missing if <10 mg/dL. *f*. Variability defined by the coefficient of variation.

BMI  =  Body Mass Index; LOS  =  Length of Stay; SpO_2_ =  Percent Oxygen Saturation; GCS  =  Glasgow Coma Scale.

**Table 3:** Unadjusted odds ratios for variables retained in the final logistic regression model predicting readmission within 48 hours of ICU discharge.

Variable	Unadjusted Odds Ratio	95% CI	Patients with Complete DataN = 469,976
**Admission Characteristics**			
** Age (per year)**			469,976 (100%)
** <70**	1.02	1.02–1.02	
** >70**	1.00	1.00–1.01	
** Elective surgery (True)** a	0.77	0.73–0.80	469,976 (100%)
** ICU Type**			469,976 (100%)
** Cardiac Medical (reference)**	1.00	–	
** Cardiothoracic Surgery**	0.95	0.88–1.03	
** Cardiovascular Surgery**	1.00	0.89–1.13	
** Medical**	1.01	0.94–1.09	
** Mixed Medical Surgical**	0.97	0.92–1.01	
** Neurological**	0.87	0.79–0.96	
** Surgical**	1.09	1.01–1.17	
** Trauma**	0.77	0.51–1.15	
** Vascular**	1.01	0.74–1.38	
** Admission Diagnosis Category** b			469,976 (100%)
** Acute Coronary Syndrome (reference)**	1.00	–	
** Acute Renal Failure**	0.89	0.67–1.16	
** Arrhythmia**	3.05	2.55–3.66	
** Asthma or Emphysema**	2.19	1.79–2.67	
** Coronary Artery Bypass Graft**	1.54	1.28–1.85	
** Cerebrovascular accident/stroke**	1.11	0.92–1.35	
** Cardiovascular (Medical)**	2.71	2.20–3.32	
** Cardiovascular (Other)**	1.54	1.25–1.90	
** Cancer**	1.02	0.78–1.34	
** Cardiac Arrest**	4.98	4.07–6.08	
** Cardiogenic Shock**	3.20	2.09–4.92	
** Chest Pain Unknown Origin**	1.09	0.86–1.39	
** Coma**	3.05	2.50–3.72	
** Diabetic Ketoacidosis**	0.37	0.27–0.50	
** Gastrointestinal Bleed**	1.46	1.21–1.76	
** Gastrointestinal Obstruction**	0.97	0.73–1.30	
** Acute Myocardial Infarction**	0.42	0.33–0.52	
** Neurologic**	1.45	1.19–1.76	
** Other (including missing)**	1.44	1.22–1.71	
** Overdose**	0.27	0.19–0.38	
** Pneumonia**	2.09	1.73–2.53	
** Respiratory (Medical/Other)**	3.85	3.23–4.58	
** Sepsis**	1.21	1.00–1.46	
** Thoracotomy**	0.58	0.43–0.80	
** Trauma**	0.54	0.43–0.68	
** Valve Disease**	0.77	0.58–1.01	
** Admit Source**			469,976 (100%)
** Floor (reference)**	1.00	–	
** Direct Admit**	1.14	1.06–1.24	
** Emergency Room**	0.91	0.87–0.96	
** Operating Room or Recovery Room**	0.78	0.74–0.83	
** Other**	1.10	1.00–1.20	
** ICU Visit number**			469,976 (100%)
** First stay of hospitalization (reference)**	1.00	–	
** At least second ICU stay**	0.04	0.02–0.05	
** BMI (per kg/m^2^)** c			
** <17**	1.00	0.94–1.07	424,316 (90.3%)
** 17**–**23**	0.96	0.94–0.97	
** >23**	1.00	0.99–1.00	
**ICU Interventions**			
**Number of lactate values in 24 hrs**			469,976 (100%)
** Zero**	1.00	–	
** At least 1**	1.26	1.17–1.35	
** ICU LOS (days)**			469,976 (100%)
** <1 day**	0.76	0.66–0.89	
** 1–2 days**	1.22	1.16–1.29	
** >2 days**	1.03	1.03–1.03	
**Last Day Labs**			
** Average Sodium (per mEq/L)**			431,570 (91.8%)
**<124 mEq/L**	1.03	0.97–1.10	
**124–138 mEq/L**	0.97	0.96–0.97	
**138–141 mEq/L**	1.05	1.03–1.07	
**>141 mEq/L**	1.06	1.05–1.07	
** Average Serum Bicarbonate (per mEq/L)**			415,625 (88.4%)
**<17 mEq/L**	1.07	1.01–1.13	
**17–25 mEq/L**	0.95	0.94–0.96	
**25–38 mEq/L**	1.04	1.03–1.05	
**>38 mEq/L**	1.01	0.97–1.05	
** Average White Blood Cell Count (per 10^3^/µL)**			418,283 (89.0%)
** <4×10^3^/µL**	0.86	0.79–0.92	
** 4–9×10^3^/µL**	1.01	1.00–1.03	
** 9–40×10^3^/µL**	1.03	1.02–1.03	
** >40×10^3^/µL**	0.96	0.93–0.99	
** Maximum Serum Creatinine (per mg/dL)**			430,885 (91.7%)
**<1 mg/dL**	1.01	0.88–1.15	
**1–2 mg/dL**	1.65	1.55–1.76	
**>2 mg/dL**	0.96	0.95–0.98	
** Average Hemoglobin (per g/dL)**			425,681 (90.6%)
**<8 g/dL**	1.14	0.98–1.32	
**8–13 g/dL**	0.93	0.91–0.94	
**>13 g/dL**	0.98	0.94–1.01	
**Last Day Physiology**			
** Heart Rate Variability** d **(per 100 units)**			463,780 (98.7%)
** <2.2**	1.18	1.04–1.35	
** 2.2–8**	0.94	0.93–0.95	
** >8**	1.01	1.00–1.01	
** Maximum Heart Rate (per bpm)**			464,494 (98.8%)
**<100 bpm**	1.00	1.00–1.01	
**100–185 bpm**	1.01	1.01–1.01	
**>185 bpm**	0.99	0.98–1.01	
** Respiratory Rate Variability** d			448,978 (95.5%)
** <0.2**	1.19	0.74–1.94	
** >0.2**	0.96	0.67–1.37	
** Maximum Diastolic Blood Pressure (per mmHg)**			456,617 (97.2%)
**<82 mmHg**	0.99	0.99–1.00	
**>82 mmHg**	1.00	1.00–1.00	
** Minimum Systolic Blood Pressure (per mmHg)**			456,637 (97.2%)
**<105 mmHg**	0.99	0.99–0.99	
**>105 mmHg**	1.00	1.00–1.01	
** Minimum Heart Rate (per bpm)**			464,494 (98.8%)
**<70 bpm**	1.00	1.00–1.01	
**70–120 bpm**	1.02	1.02–1.02	
**>120 bpm**	0.98	0.91–1.06	
** Most Recent GCS Score**			264,657 (56.3%)
** <10**	1.14	1.08–1.20	
** >10**	0.87	0.85–0.88	
** Minimum SpO_2_ (per unit)**			458,080 (97.5%)
** <87%**	1.00	0.99–1.00	
** 87–98%**	0.94	0.93–0.95	
** >98%**	1.18	1.04–1.34	
** Average Respiratory Rate (per bpm)**			452,882 (96.4%)
**<8 bpm**	1.36	0.82–2.26	
**8–12 bpm**	0.89	0.81–0.98	
**12–20 bpm**	1.04	1.03–1.05	
**20–40 bpm**	1.07	1.07–1.08	
**>40 bpm**	0.64	0.45–0.90	

a 47,231 (10.0%) missing and considered to be “False”.

b 47,231 (10.0%) missing and classified as “Other”.

c BMI considered missing if <10 or >125.

d Variability defined by the coefficient of variation.

BMI  =  Body Mass Index; LOS  =  Length of Stay; SpO_2_ =  Percent Oxygen Saturation; GCS  =  Glasgow Coma Scale.

**Figure 2:**
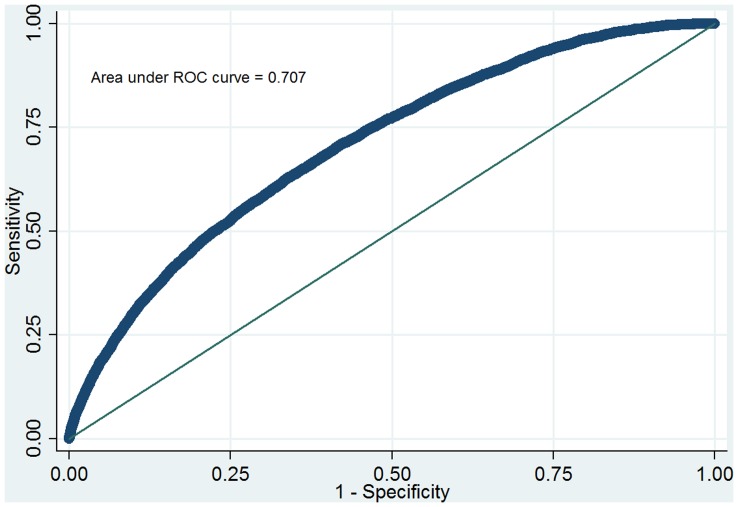
Receiver Operating Characteristic curve for the ICU Discharge Readiness Score prediction model for readmission in the validation cohort.

**Figure 3:**
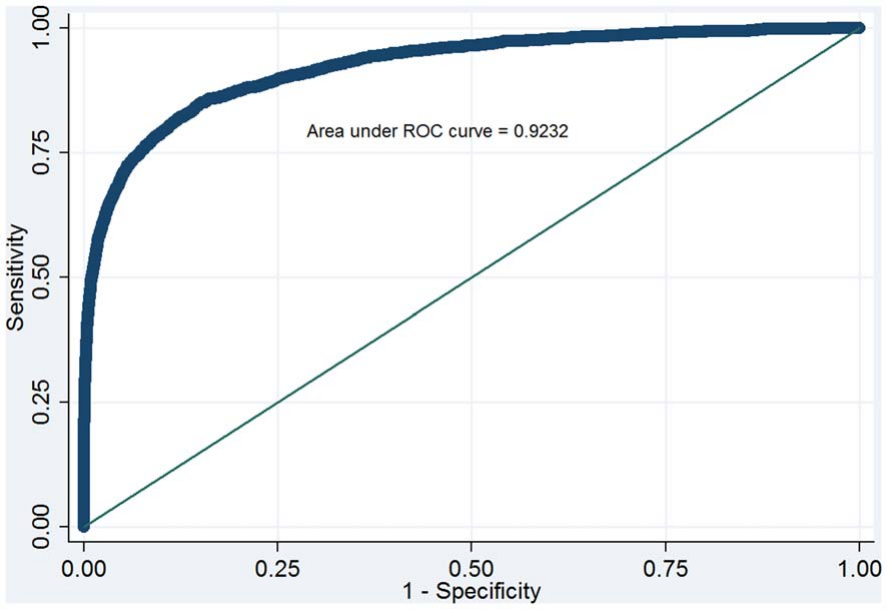
Receiver Operating Characteristic curve for the ICU Discharge Readiness Score prediction model for death in the validation cohort.

Across the multiply imputed datasets, the final readmission model produced a median auROC of 0.71 (range: 0.7058–0.7061) in the development set (N = 469,976) and 0.71(range: 0.7060–0.7068) in the validation set (N = 234,987). Figure 2 displays the median ROC curve for the validation cohort. Figure 3 displays the final model predicting death within 48 hours of ICU discharge, which produced a median auROC of 0.92 (range: 0.9227–0.9241) in the development set (N = 469,976) and 0.92 (range: 0.9226–0.9242) in the validation set (N = 234,987).

**Figure 4:**
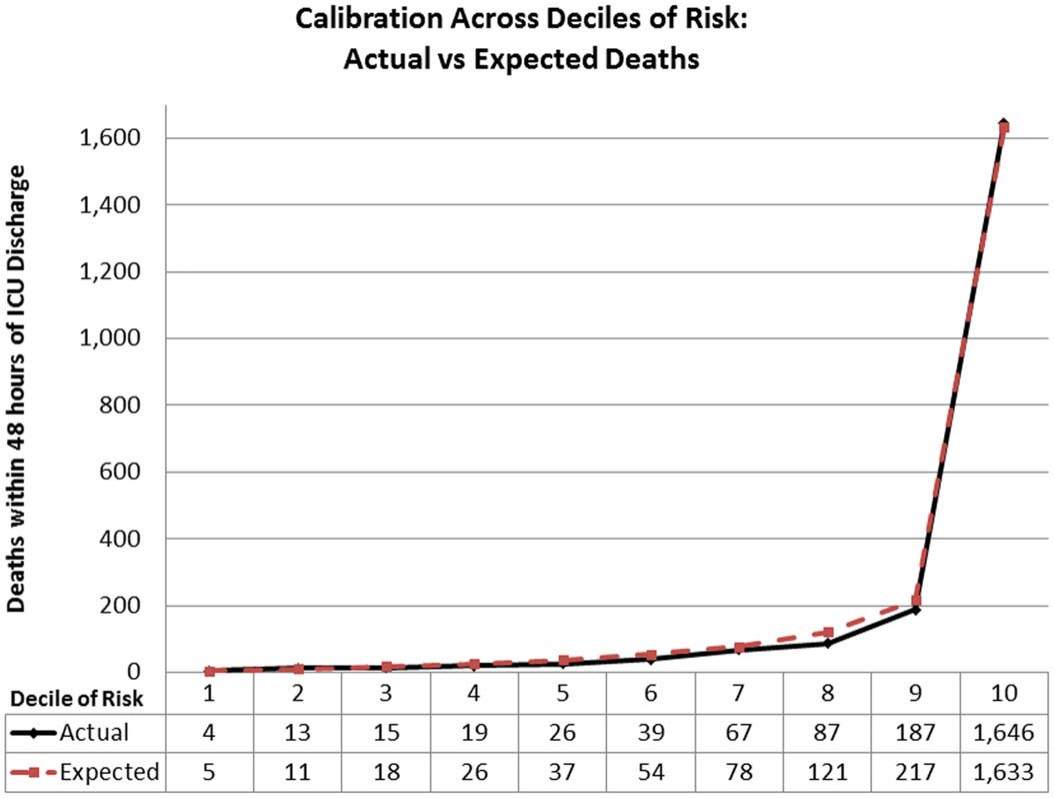
Calibration across deciles of predicted risk of death.

**Figure 5:**
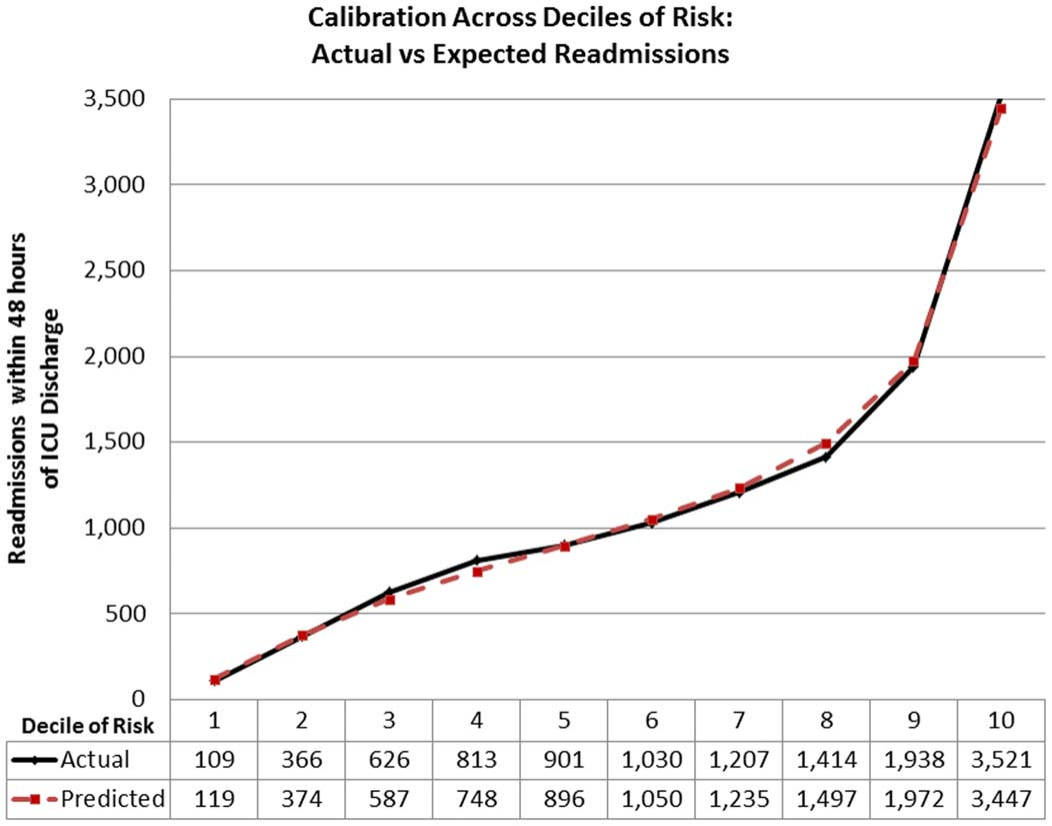
Calibration across deciles of predicted risk of readmission.

The actual to predicted ratio of readmissions within 48 hours of ICU discharge in the validation cohort was 1.0 (5,949 actual readmissions to 5,954.6 predicted). The actual to predicted ratio of deaths within 48 hours of ICU discharge in the validation cohort was 0.96 (2,103 actual deaths to 2,199.4 predicted). The median Hosmer-Lemeshow goodness-of-fit test statistics for the readmission model were: Chi2 = 17.57 (range Chi2 = 14.12–20.26; p = 0.03) and Chi2 = 7.17, (range: 4.52–7.17; p = 0.52) in the development and validation cohorts respectively. Statistically there was poor fit in both the development (median Chi2 = 53.24, p<0.01; range: 49.92–61.26) and validation cohorts (median Chi2 = 25.68, p<0.01; range: 25.40–29.26) of the mortality model. Due to the limited clinical value of statistical tests of fit in such large sample sizes [28], the calibration across deciles of risk for both models are presented in Figures 4 and 5 to provide clinical perspective to differences in actual to predicted rates. The actual to predicted rates of death and readmission across categories of hospital bed count and teaching status are presented in Table 4. Actual to predicted rates for different ICU types are presented in Table 5.

**Table 4:** Actual to expected events by hospital type.

Hospital Size(Number of beds)	N	Actual : Predicted Readmissions	Actual : Predicted Deaths
**Teaching Hospitals**			
** <100**	0	–	–
** 100–250**	765	22 : 14	10 : 12
** 250–500**	5,240	127 : 121	62 : 60
** >500**	43,319	1,116 : 1,157	479 : 439
**Non-Teaching Hospitals**			
** <100**	13,273	299 : 342	127 : 142
** 100–250**	48,765	1,226 : 1,259	457 : 477
** 250–500**	60,038	1,441 : 1,456	509 : 516
** >500**	63,587	1,691 : 1,633	459 : 510

**Table 5:** Calibration across seven different ICU types.

ICU Type	Actual Deaths	Predicted Deaths	SMR	Actual Readmits	Predicted Readmits	A:P Ratio
**Cardiac Medical**	425	430.1	0.99	1,214	1,195.0	1.02
**Cardiovascular or Cardiothoracic Surgery**	209	193.9	1.08	613	599.0	1.02
**Medical**	226	222.7	1.01	550	491.7	1.12
**Mixed Medical-Surgical**	1,023	1,117.0	0.92	2,762	2,883.2	0.96
**Neurological**	108	81.8	1.32	250	248.3	1.01
**Surgical**	109	108.3	1.01	501	484.6	1.03
**Trauma** a	3	1.78	–	15	10.7	–

a Too few patients to reliably calculate.

SMR  =  standardized mortality ratio; A:P  =  actual to predicted.

The median sensitivity, specificity, positive predictive value (PPV) and negative predictive value (NPV) at various predicted probabilities of early death and readmission after ICU discharge are presented in Table 6. Due to the relatively low prevalence of each outcome, both models tend to have high negative predictive values and low positive predictive values. 17% of ICU discharges went to a step-down unit compared to 83% to a general ward. Using a robust variance estimator clustered by ICU, the unadjusted OR for death was 41% higher (p<0.03) if discharged to a step-down unit, but this association was reduced once adjusted for the predicted log-odds of death (OR = 1.31; p = 0.02). The unadjusted odds of readmission were 35% lower in a step-down unit and remained 36% lower after adjusting for the predicted log odds of readmission (p<0.001 for both).

**Table 6:** Performance of the ICU Discharge Readiness Score models in the validation cohorts.

Predicted Probability	Sensitivity (%)		Specificity (%)		PPV^a^ (%)		NPV^b^ (%)	
Death/Readmission	Death	Readmission	Death	Readmission	Death	Readmission	Death	Readmission
**0.1%**	98.38	99.88	32.53	3.35	1.30	2.61	99.96	99.91
**1%**	82.26	96.42	87.23	19.30	5.50	3.01	99.82	99.52
**2.5%**	69.19	65.78	95.38	62.84	11.90	4.40	99.71	98.61
**5%**	58.30	29.64	98.00	90.31	20.83	7.36	99.62	98.02
**10%**	47.27	5.66	99.15	99.01	33.52	12.92	99.52	97.58
**25%**	33.62	0	99.73	99.99	53.32	0	99.40	97.47
**50%**	22.63	0	99.91	100	68.99	–	99.31	97.47

a PPV  =  Positive Predictive Value.

b NPV  =  Negative Predictive Value.

**Figure 6:**
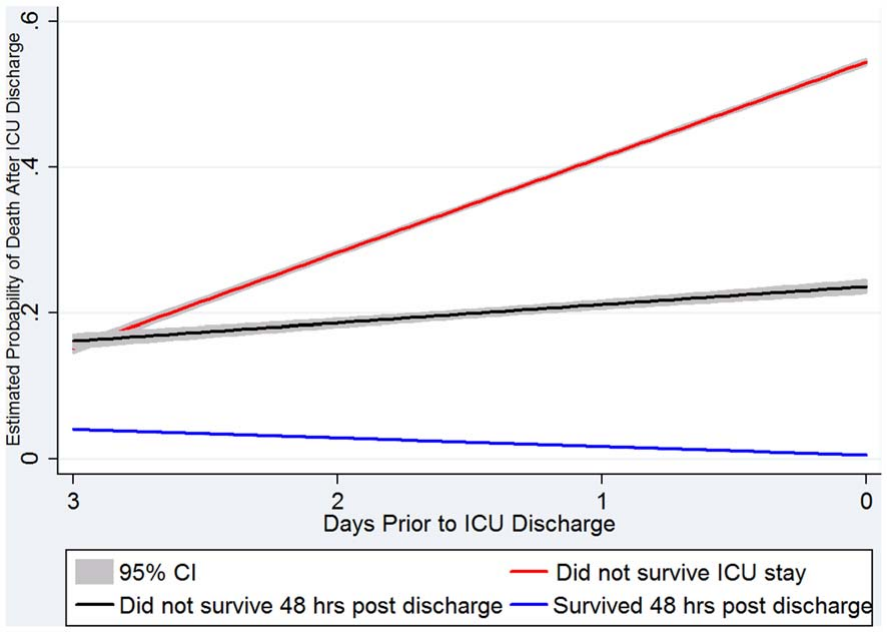
Average change in estimated probability of death across last four days in the ICU.

In order to simulate performance in a real-time environment, fitted values were generated for patients between one and four days prior to ICU discharge in the validation cohort. Table 7 shows the median and IQR for the predicted risk of death and readmission in patients who did not have a complication within 48 hours of ICU discharge, had a complication within 48 hours after discharge and those who did not survive their ICU stay. The average change in predicted risk of death across the last four ICU days is graphically represented in Figure 6.

**Table 7:** Predicted risk of death and readmission at 24 hour intervals in up to the last four days in the ICU.

Percent Risk in Patient Subgroups	Days Prior to ICU Discharge^a^				Rate of Change	
	3	2	1	0	Average per Day^b^	95% CI
**Risk of Death Model**						
** Survived 48 hours after ICU discharge, Median % (IQR)**	0.80(0.26–2.96)	0.55(0.19–1.83)	0.30(0.11–0.89)	0.19(0.07–0.48)	−1.18	−1.21 – −1.16^c^
** Did not survive 48 hours after ICU discharge, Median % (IQR)**	7.01(2.02–23.77)	6.44(1.83–24.16)	6.29(1.73–26.63)	8.82(1.73–43.53)	2.47	1.98 – 2.97^d^
** Did not survive the ICU stay,** **Median % (IQR)**	11.11(2.87–36.50)	12.51(3.07–41.41)	16.61(3.88–53.16)	69.51(25.20–94.74)	13.08	12.81 – 13.34^e^
**Risk of Readmission Model**						
** No readmission within 48 hours after ICU discharge, Median % (IQR)**	2.71(1.63–4.40)	2.50(1.51–3.98)	2.19(1.32–3.44)	2.04(1.24–3.11)	−0.31	−0.32 – −0.31^c^
** Readmitted within 48 hours after ICU discharge, Median % (IQR)**	4.21(2.63–6.93)	3.82(2.43–6.50)	3.42(2.21–5.76)	3.30(2.15–5.52)	−0.35	−0.39 – −0.31^d^
** Did not survive the ICU stay,** **Median % (IQR)**	4.08(2.28–7.07)	4.13(2.19–6.99)	4.14(2.17–7.14)	4.20(2.23–7.37)	0.07	0.03 – −0.10^e^

a Day 0 =  predictions generated at the time of ICU discharge; Day 1 =  predictions generated 24 hours prior to discharge; Day 2 =  predictions generated 48 hours prior to discharge; Day 3 =  predictions generated 72 hours prior to discharge.

b The average change in predicted risk (%) across the last four ICU days using linear regression with a robust variance estimator clustered by patient across ICU days.

c 600,252 observations.

d 9,830 observations.

e 40,801 observations. All p-values <0.01.

## Discussion

The rate of readmission and death within 48 hours of ICU discharge was approximately 2.5% and 0.9%, respectively, in this heterogeneous critically ill population from 219 hospitals and 402 ICUs. Predictive algorithms for death and readmission within 48 hours of ICU discharge developed from this large dataset had excellent and moderate discrimination, respectively, and both calibrated well across risk strata. Despite efforts to limit the number of variables in the predictive models, optimal performance required more than 20 variables, a number that significantly exceeds human processing capacity but relatively simple for a computer to analyze. We intend for these models to be calculated automatically on a continuous basis for patients all patients eligible for consideration for ICU discharge. Although predictions would only be generated for patients with complete data available, the use of multiply imputed data suggests the discrimination of the models are robust to the missing data patterns observed in this cohort. We speculate that post discharge risk estimates might be helpful in discharge decision making given the time devoted to this activity and the adverse consequences of delaying discharge or sending patients out prematurely.

Prior studies of post discharge adverse events have generally focused on readmission as the endpoint of interest or have used a combined endpoint of readmission or death. [13], [17], [18] This appeared to be a rational approach because it seemed plausible that both unexpected readmission and death were due to post discharge clinical deterioration. Moreover, these smaller studies lacked sufficient numbers of post discharge deaths to analyze the two endpoints separately. The auROC in these previous studies ranged between 0.62 and 0.74, and calibration was generally poor. [13], [17], [18] It should be noted that the validation arm for the SWIFT score was a cohort of patients from a different institution, which is inherently more difficult to achieve good calibration than during internal validation such as ours. [13] In our analysis, the auROC for the readmission model was similar to those described in prior studies, although calibration was excellent, especially considering it is rare to observe a positive goodness of fit test with large sample size. [28] Moreover, the model performed well across different types of ICUs and across hospitals of different sizes and teaching status which we hypothesize is a function of the broad population from which it was generated. The moderate discrimination of our model suggests that a significant proportion of factors contributing to ICU readmission are not clinically observable at the time of ICU discharge. These may include the quality of care provided at the lower acuity (receiving) unit and provider variability in assessing readmission need. There also appears to be a lower likelihood of readmission when the lower acuity unit is a step-down unit compared to a general ward. We attempted to minimize the influence of care provided after discharge by limiting the outcome to within 48 hours of ICU discharge, but despite this design, latent variables appear to affect readmission risk. Surprisingly, this limitation was not a major factor when predicting death shortly after ICU discharge.

With 469,976 patients and over 4,389 deaths in the development cohort, we were able to model death as a distinct outcome; the differences between the two models were striking. The risk of death model had extremely high discrimination reflected in an auROC of 0.92. Although this seems implausibly high, close examination of the predictors included in the model indicated very strong relationships with death after discharge (Table 2). Although the explanation for superior discrimination is unknown, we speculate that the endpoint of death is less likely to be influenced by subjective decisions driven by social and political factors. In support of this hypothesis, the model predicting death requires fewer static patient characteristics such as, admission diagnosis, type of ICU and admission source than the readmission model. It also is likely that ICU readmission criteria vary among the 219 hospitals included in this cohort. We also noted that many physiologic variables, when significantly abnormal, were associated with a high risk of death, but a low risk of readmission. For example, the risk of death increased linearly for white blood cell counts above 9,000 cells/µL, but the risk of readmission declined above 40,000 cells/µL (Tables 2 and 3).

Although the models were developed on data available on the day of discharge, the secondary validation we performed using patient data from one, two and three days prior to ICU discharge suggest that the model might provide clinical utility. The model for death discriminated between patients who were stable enough for ICU discharge from those who were not. Despite excluding all patients who died in the ICU during model development, the predicted risk of death in these patients was dramatically higher than those who were discharged without complication, even up to four days prior to discharge (Table 7 and Figure 6).

Ideally these models can be incorporated into an electronic clinical decision support tool for use in ICU discharge planning, with a goal of improving patient safety and increasing ICU throughput. Zimmerman and Kramer have previously suggested that patients admitted to the ICU with a low predicted risk of active treatments, referred to as “low-risk monitor” patients, may not necessarily require an ICU admission. [29] Using the *e*RI database from 2008, Lilly et al. reported that approximately 40% of ICU admissions had low day 1 mortality risk and received no major therapies. [24] The trends observed in Table 7 appear to support this notion that a substantial proportion of ICU days are attributable to patients not requiring intensive care treatments. In translating these into CDS tools, we believe that due to the differences in performance, clinicians should view the risk of readmission as complementary information to the more accurate predictions generated from the model predicting death. We also believe there is value in defining multiple categories of risk, rather than trying to use a single threshold to determine whether a patient is ready for discharge. For example, if a threshold for ‘low risk’ of death is defined as a prediction under 0.1%, greater value can be derived from the high NPV. With this threshold, 32% of the validation cohort is defined as ‘low risk’ at the time of discharge with a false negative rate of 0.046%. For readmission, if ‘low risk’ is defined as below 1% predicted probability of readmission, 19% of the validation cohort was below this threshold and the false negative rate was only 0.5% (Table 6). The simulation study also identified many patients with low risk of death and readmission who stayed in ICU for additional days. 21% and 18% of all patients discharged would have been classified as ‘low risk’ of death and readmission, respectively 24 hours prior to their actual ICU discharge.

Prospective validation is clearly warranted, but there appears to be an opportunity to use this tool to support clinical programs aimed at reducing unnecessary ICU days. As Zimmerman and Kramer point out, “Improved resource use and reduced costs might be achieved by strategies to provide care for these patients on floors or intermediate care units.” [29] At the other end of the risk spectrum, our data indicate that more than 2% of discharges either died or required readmission. If ‘high risk’ were defined as greater than 5% predicted risk of death, 2.4% of those discharged would have been identified as ‘high risk’ at the time of discharge, with 22% of these dying in the subsequent 48 hours. We speculate that some of these patients were not recognized as being at high risk for death after discharge and providing this information may help clinicians decide to prolong the ICU stay in these patients. Although we could not confirm this, it seems plausible the unexpected deaths represented sudden cardiorespiratory arrest whereas a more gradual deterioration may often be required for a readmission to occur. With an auROC of 0.92, these deaths were predictable and potentially preventable. Given this is a very small proportion of ICU discharges, considering a prolonged ICU stay to mitigate risks may be a reasonable choice.

This study has several important limitations. Calculation of the ICU Discharge Readiness Score is relatively complex and cannot be performed manually, although it can be easily programmed to provide electronic clinical decision support. As a retrospective study, the accuracy and completeness of data is limited by the quality of documentation in the clinical information system, which likely is less accurate than data collected in a tightly controlled clinical trial. This approach was utilized to create a very large sample size with many sentinel events (death and readmission) and to include data from many different institutions. Several strategies were employed to minimize artifacts. Vital signs in the *e*RI database are archived as 5-minute medians (from the 1-minute averages received from bedside monitors via interfaces). Many of the variables used 24 hour averages to minimize the impact of outlier data. Patients with documented care limitations were excluded from analysis because death in some of these patients was seen as their expected outcome, and readmission would have been inappropriate. It is possible that some patients with care limitations lacked appropriate documentation and were included in the analysis.

In general, data completeness was reasonable, especially considering missing data tends to be more common on the day of ICU discharge when patients are more stable than earlier in the ICU stay. Most variables were available during the final 24 hours before ICU discharge with the exception of GCS scores. The high proportion of missing GCS values on the day of discharge may be due to variability in the use of GCS scores in some ICU populations and the tendency of *e*ICU Programs to use the remote management software for population management, rather than focusing on comprehensive documentation for the medical record. Some participating health systems have not implemented an interface to import nursing flowsheet data from the primary electronic medical record, which results in absence of GCS values. As shown by other investigators (e.g., SWIFT) and confirmed in this analysis, GCS is an important predictor of death and readmission after ICU discharge.(12) Introduced by Rubin, the method of choice for reducing bias introduced by missing data is through multiple imputation.[25]–[27] Development and validation on multiply imputed data provides confidence that our models are robust to variations in real-world documentation practices. Because our ultimate goal is to have accurate predictive models that can be used in large numbers of patients, rather than establishing causality, decisions regarding which data elements are used in the final models must balance data availability, data reliability and model performance considerations. High degrees of collinearity (e.g., use of both average heart rate and highest heart rate) were allowed because the goal was not to determine the independent effect of factors such as, average heart rate, but to provide the best estimate of the risk of post-discharge death and readmission. As a result, it is not possible to clinically interpret the adjusted odds ratios for many of the variables (data not shown). Also, the list of variables predictive of readmission and death presented is not exhaustive since variables were only retained if they resulted in improved performance of the models. Lastly, these models should be viewed as tools to support clinical workflow rather than replace clinical judgment. We believe any successful program for improving ICU discharge planning will require establishing standardized processes to reduce the variability with which these decisions are generally made today.

### Conclusion

Death and readmission within 48 hours of ICU discharge are clinically distinct outcomes, requiring independent predictive models. The predictive models for death and readmission calibrate well across deciles of risk and exhibit excellent and moderate discrimination respectively. The model predicting the risk of death accurately discriminated between patients who would and would not experience a complication as early as four days prior to ICU discharge. We speculate that these predictive models may improve ICU discharge planning if incorporated into a clinical decision support application that can provide actionable information to ICU clinicians.

## Supporting Information

Table S1 Variables evaluated for inclusion in ICU Discharge Readiness Score models.
(DOCX)

Table S2 APACHE Admission Diagnosis Groupings.
(DOCX)
