# Supplementary material for: Readmissions and Death after ICU Discharge: Development and Validation of Two Predictive Models
Source: PLoS One. 2012 Nov 7;7(11):e48758. doi: 10.1371/journal.pone.0048758 (PMC3492441; doi:10.1371/journal.pone.0048758)
Supplement: Table S1 — Variables evaluated for inclusion in ICU Discharge Readiness Score models. (DOCX) [file pone.0048758.s001.docx]

Table S1. Variables evaluated for inclusion in ICU Discharge Readiness Score models.

| Admission Characteristics: |
| --- |
| ICU Visit number |
| Age |
| ICU Length of Stay*^a^* |
| Hospital Length of Stay*^a^* |
| Admission Source |
| Admission Diagnosis*^b^* |
| Operative Admission Diagnosis*^c^* |
| Elective Surgical Admission Diagnosis*^c^* |
| APACHE IV Score |
| Body Mass Index (BMI) |
| Ward type |
| Gender |
| ICU Interventions: |
| Time since central line removal |
| Airway status |
| Ventilation status |
| Time since continuous infusion |
| Time since ventilation |
| Lactate count*^d^* |
| Hemoglobin count*^d^* |
| FiO_2_ worst*^e^* |
| Last day laboratory values: |
| Anion Gap (maximum) |
| Serum Creatinine (maximum) |
| White Blood Cell Count (average) |
| Serum Bicarbonate (average) |
| Hemoglobin (average) |
| Hemoglobin (minimum) |
| Hemoglobin (coefficient of variation) |
| Bilirubin (average) |
| Serum Lactate*^f^* |
| Blood Glucose (average) |
| Blood Glucose (coefficient of variation) |
| Blood Glucose (maximum) |
| Hypoglycemia |
| Serum Sodium (average) |
| pH (average)*^g^* |
| Serum Potassium (average) |
| Serum Potassium (minimum) |
| Last day physiology: |
| Respiratory Rate (average) |
| Respiratory Rate (maximum) |
| Respiratory Rate (minimum) |
| Respiratory Rate (coefficient of variation) |
| SpO_2_ (minimum) |
| SpO_2_ (average) |
| SpO_2_ (coefficient of variation) |
| Most recent GCS |
| Change in GCS over last 24 hours |
| Heart Rate (average) |
| Heart Rate (minimum) |
| Heart Rate (maximum) |
| Heart Rate (coefficient of variation) |
| Systolic Blood Pressure (average) |
| Systolic Blood Pressure (minimum) |
| Systolic Blood Pressure (maximum) |
| Systolic Blood Pressure (coefficient of variation) |
| Diastolic Blood Pressure (average) |
| Diastolic Blood Pressure (minimum) |
| Diastolic Blood Pressure (maximum) |
| Diastolic Blood Pressure (coefficient of variation) |
| Temperature (average) |

*a.* ICU and Hospital length of stay capped at 30 days. *b*. Missing classified as “Other”. *c*. Missing classified as “False”. *d*. Number of lab draws in the preceding 24 hours. *e*. Considered equal to room air (21%) if missing. *f*. Considered 0 if missing. *g*. Considered normal if missing.

APACHE = Acute Physiology and Chronic Health Evaluation; FiO_2_ = fraction of inspired oxygen; SpO_2_ = Percent Oxygen Saturation.
