# Supplementary material for: Readmissions and Death after ICU Discharge: Development and Validation of Two Predictive Models
Source: PLoS One. 2012 Nov 7;7(11):e48758. doi: 10.1371/journal.pone.0048758 (PMC3492441; doi:10.1371/journal.pone.0048758)
Supplement: Table S2 — APACHE Admission Diagnosis Groupings. (DOCX) [file pone.0048758.s002.docx]

Table S2. APACHE Admission Diagnosis Groupings

| **Acute Coronary Syndrome** | “Angina, unstable (angina interferes w/quality of life or meds are tolerated poorly)" |
| --- | --- |
| **Acute Myocardial Infarction** | "Infarction, acute myocardial (MI)"; MI admitted > 24 hrs after onset of ischemia" |
| **Chest Pain Unknown Origin** | "Chest pain, atypical (noncardiac chest pain)"; "Chest pain, epigastric"; "Chest pain, musculoskeletal"; "Chest pain, respiratory"; Chest pain, unknown origin" |
| **Cardiogenic Shock** | **“**Shock, cardiogenic" |
| **Cardiovascular (Other)** | **“**Angina, stable (asymp or stable pattern of symptoms w/meds)"; "Anomaly, cardiac congenital"; "Arteriovenous malformation, surgery for"; "Atrial Septal Defect (ASD) Repair"; "Cardiovascular surgery, other"; "Congenital Defect Repair (Other)"; CVOther ="Contusion, myocardial (include r/o)"; "Efffusion, pericardial"; "Endocarditis"; "Hypertension-pulmonary, primary/idiopathic"; "Monitoring, hemodynamic (pre-operative evaluation)"; "Pericardial effusion/tamponade"; "Pericardiectomy (total/subtotal)"; "Pericarditis"; "Tamponade, pericardial"; "Thrombus, arterial"; "Vascular medical, other"; "Vascular surgery, other" |
| **Cardiovascular (Medical)** | **“**Cardiovascular medical, other" |
| **Cardiac Arrest** | **“**Cardiac arrest (with or without respiratory arrest; for respiratory arrest see Respiratory System)" |
| **Arrhythmia** | "Ablation or mapping of cardiac conduction pathway"; "Defibrillator, automatic implantable cardiac; insertion of"; "Rhythm disturbance (atrial, supraventricular)"; "Rhythm disturbance (conduction defect)"; "Rhythm disturbance (ventricular)" |
| **Coronary Artery Bypass Graft** | "CABG alone, coronary artery bypass grafting"; "CABG alone, redo"; "CABG redo with other operation"; "CABG redo with valve repair/replacement"; "CABG with aortic valve replacement"; "CABG with double valve repair/replacement"; "CABG with mitral valve repair"; "CABG with mitral valve replacement"; "CABG with other operation"; "CABG with pulmonic or tricuspid valve repair or replacement ONLY."; "CABG with single valve repair/replacement"; "CABG, minimally invasive; mid-CABG" |
| **Valve Disease** | "Aortic and Mitral valve replacement"; "Aortic valve replacement (isolated)"; "Mitral valve repair"; "Mitral valve replacement"; "Papillary muscle rupture"; "Pulmonary valve surgery"; "Tricuspid valve surgery"; "Valve, double; repair/replacement"; "Valve, redo, single"; "Valve, single; repair/replacement"; "Valve, triple; repair/replacement" |
| **Pneumonia** | "Pneumonia, aspiration"; "Pneumonia, bacterial"; "Pneumonia, fungal"; "Pneumonia, other"; "Pneumonia, parasitic (i.e., Pneumocystic pneumonia)"; "Pneumonia, viral" |
| **Respiratory (Medical/Other)** | "Apnea, sleep"; "Apnea-sleep; surgery for (i.e., UPPP - uvulopalatopharyngoplasty)"; "ARDS-adult respiratory distress syndrome, non-cardiogenic pulmonary edema"; "Arrest, respiratory (without cardiac arrest)"; "Atelectasis"; "Biopsy, open lung"; "Effusions, pleural"; "Embolus, pulmonary"; "Guillain-Barre syndrome"; "Hemorrhage/hemoptysis, pulmonary"; "Hemothorax"; "Obstruction-airway (i.e., acute epiglottitis, post-extubation edema, foreign body, etc)"; "Pneumothorax"; "Respiratory - medical, other"; "Restrictive lung disease (i.e., Sarcoidosis, pulmonary fibrosis)"; "Tracheostomy"; "Weaning from mechanical ventilation (transfer from other unit or hospital only)" |
| **Asthma or Emphysema** | "Asthma"; "Emphysema/bronchitis" |
| **Gastrointestinal Bleed** | "Bleeding, GI from esophageal varices/portal hypertension"; "Bleeding, GI-location unknown"; "Bleeding, lower GI"; "Bleeding, upper GI"; "Bleeding-lower GI, surgery for"; "Bleeding-other GI, surgery for"; "Bleeding-upper GI, surgery for"; "Bleeding-variceal, surgery for (excluding vascular shunting-see surgery for portosystemic shunt)"; "GI perforation/rupture"; "GI perforation/rupture, surgery for"; "Hemorrhage, intra/retroperitoneal"; "Ulcer disease, peptic" |
| **Gastrointestinal Obstruction** | "GI obstruction"; "GI obstruction, surgery for (including lysis of adhesions)" |
| **Cerebrovascular Accident/Stroke** | "CVA, cerebrovascular accident/stroke"; "Hemorrhage/hematoma, intracranial"; "Hemorrhage/hematoma-intracranial, surgery for"; "Hypertension, uncontrolled (for cerebrovascular accident-see Neurological System)"; "Subarachnoid hemorrhage/arteriovenous malformation"; "Subarachnoid hemorrhage/intracranial aneurysm"; "Subarachnoid hemorrhage/intracranial aneurysm, surgery for" |
| **Neurologic** | "Abscess, neurologic"; "Biopsy, brain"; "Hydrocephalus, obstructive"; "Neoplasm, neurologic"; "Neoplasm-cranial, surgery for (excluding transphenoidal)"; "Neoplasm-spinal cord, surgery or other related procedures"; "Neurologic medical, other"; "Neuromuscular medical, other"; "Palsy, cranial nerve"; "Seizures (primary-no structural brain disease)"; "Seizures-intractable, surgery for" |
| **Coma** | "Coma/change in level of consciousness (for hepatic see GI, for diabetic see Endocrine, if related to cardiac arrest, see CV)"; "Nontraumatic coma due to anoxia/ischemia" |
| **Overdose** | "Overdose, alcohols (bethanol, methanol, ethylene glycol)"; "Overdose, analgesic (aspirin, acetaminophen)"; "Overdose, antidepressants (cyclic, lithium)"; "Overdose, other toxin, poison or drug"; "Overdose, sedatives, hypnotics, antipsychotics, benzodiazepines"; "Overdose, self-inflicted"; "Overdose, street drugs (opiates, cocaine, amphetamine)"; "Toxicity, drug (i.e., beta blockers, calcium channel blockers, etc.)" |
| **Sepsis** | "Sepsis, cutaneous/soft tissue"; "Sepsis, GI"; "Sepsis, gynecologic"; "Sepsis, other"; "Sepsis, pulmonary"; "Sepsis, renal/UTI (including bladder)"; "Sepsis, unknown" |
| **Acute Renal Failure** | "Renal failure, acute"; "Renal obstruction" |
| **Diabetic Ketoacidosis** | "Diabetic hyperglycemic hyperosmolar nonketotic coma (HHNC)"; "Diabetic ketoacidosis" |
| **Trauma** | "Abdomen only trauma"; "Abdomen/extremity trauma"; "Abdomen/face trauma"; "Abdomen/multiple trauma"; "Abdomen/pelvis trauma"; "Abdomen/spinal trauma"; "Chest thorax only trauma"; "Chest/abdomen trauma"; "Chest/extremity trauma"; "Chest/face trauma"; "Chest/multiple trauma"; "Chest/pelvis trauma"; "Chest/spinal trauma"; "Chest/thorax only trauma"; "Extermity only trauma"; "Extremity only trauma, surgery for"; "Extremity/face trauma"; "Extremity/face trauma, surgery for"; "Extremity/multiple trauma"; "Extremity/multiple trauma, surgery for"; "Face only trauma"; "Face only trauma, surgery for"; "Face/multiple trauma"; "Face/multiple trauma, surgery for"; "Facial surgery (if related to trauma, see Trauma)"; "Head only trauma"; "Head/abdomen trauma"; "Head/chest trauma"; "Head/extremity trauma"; "Head/face trauma"; "Head/multiple trauma"; "Head/pelvis trauma"; "Head/spinal trauma"; "Pelvis/extremity trauma"; "Pelvis/face trauma"; "Pelvis/hip trauma"; "Pelvis/multiple trauma"; "Pelvis/spinal trauma"; "Spinal cord only trauma"; "Spinal/extremity trauma"; "Spinal/face trauma"; "Spinal/multiple trauma"; "Trauma medical, other"; "Trauma surgery, other" |
| **Cancer** | "Cancer, colon/rectal"; "Cancer, esophageal"; "Cancer, laryngeal"; "Cancer, lung"; "Cancer, oral"; "Cancer, oral/sinus, surgery for"; "Cancer, other GI"; "Cancer, pancreatic"; "Cancer, stomach"; "Cancer, tracheal"; "Cancer-colon/rectal, surgery for (including abdominoperineal resections)"; "Cancer-esophageal, surgery for (abdominal approach)"; "Cancer-laryngeal/tracheal, surgery for"; "Cancer-other GI tract, surgery for (ie., hepatoma, gallbladder etc.)"; "Cancer-small intestinal, surgery for"; "Cancer-stomach, surgery for"; "Leukemia, acute lymphocytic"; "Leukemia, acute myelocytic"; "Leukemia, chronic lymphocytic"; "Leukemia, chronic myelocytic"; "Leukemia, other" |
| **Thoracotomy** | "Thoracotomy for benign tumor (ie. mediastinal chest wall mass, thymectomy)"; "Thoracotomy for bronchopleural fistula"; "Thoracotomy for esophageal cancer"; "Thoracotomy for lung cancer"; "Thoracotomy for lung reduction" \| admissiondiagnosis=="Thoracotomy for other malignancy in chest"; "Thoracotomy for other reasons"; "Thoracotomy for pleural disease"; "Thoracotomy for thoracic/respiratory infection" |
| **Other** | All other APACHE admission diagnoses |

APACHE = Acute Physiology and Chronic Health Evaluation
